# Analytical and Clinical Performance of the Lumipulse^®^ *G* GFAP Assay in CSF samples

**DOI:** 10.1002/alz70856_096797

**Published:** 2025-12-24

**Authors:** Hisashi Nojima, Mai Yamamoto, Jo Kamada, Tomohiro Hamanaka, Katsumi Aoyagi

**Affiliations:** ^1^ FUJIREBIO Inc., Hachioji, Tokyo, Japan

## Abstract

**Background:**

Glial fibrillary acidic protein (GFAP) has been shown to be a reliable biomarker for detecting neurological disorders. Recently, we developed the Lumipulse *G* GFAP plasma assay, which is a commercially available diagnostic tool. In this study, we evaluated this assay in cerebrospinal fluid (CSF) samples.

**Method:**

The LUMIPULSE system is a fully automated chemiluminescent enzyme immunoassay (CLEIA) platform that utilizes specialized cartridges to process samples within 30 minutes. The assay, which employs a pair of proprietary monoclonal antibodies targeting GFAP, was evaluated for clinical performance using 30 CSF samples from patients diagnosed with Alzheimer's disease (AD), mild cognitive impairment (MCI), and cognitively unimpaired (CU) individuals, with 10 samples from each group. For GFAP measurement in CSF, samples were diluted 10 times using a Lumipulse sample diluent. In addition, levels of β‐amyloid 1‐40 (Aβ40), β‐amyloid 1‐42 (Aβ42), and pTau181 were simultaneously measured.

**Result:**

The Lumipulse *G* GFAP assay significantly differentiated (*p* < 0.05) between the amyloid accumulation and non‐amyloid accumulation groups, as classified by the CSF Aβ test. Furthermore, GFAP showed a moderate correlation with pTau181 (*r* = 0.588), as determined by Spearman's rank correlation coefficient. Moreover, receiver operating characteristic (ROC) analysis was performed to determine the performance of GFAP in distinguishing amyloid‐positive and amyloid‐negative subjects, and the area under the curve (AUC) was 0.72 (0.50‐0.94).

**Conclusion:**

This study demonstrates that the Lumipulse *G* GFAP assay, when applied to CSF samples, can potentially differentiate AD from non‐AD cases. These findings suggest that GFAP can be a valuable biomarker for AD, providing additional diagnostic information alongside traditional biomarkers such as Aβ40, Aβ42 and pTau181. However, since GFAP may also be elevated in other neurological disorders beyond AD, further investigation into these conditions is required.

